# Integrative oncology in clinical practice, research, and education: Middle-Eastern and global perspectives

**DOI:** 10.1007/s00520-026-10647-5

**Published:** 2026-04-22

**Authors:** Eran Ben-Arye, Tido von Schoen-Angerer, Maryam Rassouli, Gülbeyaz Can, Mohamed Hablas, Suha Omran, Konstantina Stylianou, Malak Rita Hajji, Neil Arun Nijhawan, Ibtisam Ghrayeb, Nahla Gafer, Georg Seifert, Jianping Liu, Alain Toledano, Massimo Bonucci, Ricardo Ghelman, Nina Fuller-Shavel, Rammanohar Puthiyedath, Noah Samuels, Lorenzo Cohen

**Affiliations:** 1 Ruth and Bruce Rappaport Faculty of Medicine, Technion-Israel Institute of Technology, Haifa, Israel; 2 Middle East Research Group in Integrative Oncology (MERGIO), Within Middle East Cancer Consortium (MECC), Haifa, Israel; 3 Integrative Oncology Program, The Oncology Service, Lin-Carmel-Zebulun Medical Centers, 35 Rothschild St., Haifa, Israel; 4 Society for Integrative Oncology (SIO) Regional Ambassador to Europe and the Middle East, Haifa, Israel; 5 International Federation of Anthroposophic Medical Associations IVAA, Brussels, Belgium; 6 Cancer Research Center, Shahid Beheshti University of Medical Sciences, Tehran, Iran; 7 School of Nursing, College of Health Sciences, University of Nizwa, Nizwa, Sultanate of Oman; 8 Florence Nightingale Nursing Faculty, Istanbul University - Cerrahpasa, Istanbul, Turkey; 9 Palliative Care Services, Gharbia Cancer Society, Tanta, Egypt; 10 Faculty of Nursing/WHO Collaborating Center, Jordan University of Science and Technology, Irbid, Jordan; 11 German Oncology Center, Limassol, Cyprus; 12 ONCORAD Healthcare Group, Casablanca, Morocco; 13 Burjeel Medical City, Abu Dhabi, UAE; 14 Gulf Medical University, Ajman, United Arab Emirates; 15 Makassed Hospital, East Jerusalem, Palestine; 16 Palliative Care Unit, Khartoum Oncology Hospital, Khartoum, Sudan; 17 Charité Competence Center for Traditional and Integrative Medicine (CCCTIM), Charité - Universitätsmedizin Berlin, Freie Universität Berlin, Humboldt-Universität Zu Berlin, and Berlin Institute of Health, Berlin, Germany; 18 Centre for Evidence-Based Chinese Medicine, Beijing University of Chinese Medicine, Beijing, China; 19 Rafael Institute, Integrative Medicine Center, Levallois-Perret, Paris France; 20 Integrative Oncology Project, Department Oncology, S. Andrea University Hospital, Rome, Italy; 21 Brazilian Academic Consortium for Integrative Health, CABSIN, Rio de Janeiro, Brazil; 22 British Society for Integrative Oncology (BSIO), Midhurst, UK; 23 Amrita School of Ayurveda, Amrita Vishwa Vidyapeetham, Amritapuri, India; 24 Center for Integrative Complementary Medicine, Shaare Zedek Medical Center, Faculty of Medicine, Hebrew University of Jerusalem, Jerusalem, Israel; 25 Department of Palliative, Rehabilitation, & Integrative Medicine, The University of Texas MD Anderson Cancer Center, Houston, TX USA; 26 Multidisciplinary Center for Integrative Medicine, Geneva University Hospitals, Geneve, Switzerland; 27 Hartmann Oncology Radiotherapy Group, Hartmann Radiotherapy Institute, Levallois-Perret, France; 28 Department of Integrative Medicine, Conservatoire National Des Arts Et Metiers, Paris, France; 29 Humanitas Consortium University, Rome, Italy; 30 ARTOI Foundation, Rome, Italy; 31 Department of Medicine On Primary Health Care, Faculty of Medicine, Federal University of Rio de Janeiro, Rio de Janeiro, Brazil; 32 National Centre for Integrative Oncology (NCIO), Reading, UK; 33 Integrative Cancer Care, Synthesis Clinic, Reading, UK

**Keywords:** Integrative oncology, Traditional medicine, Middle-East, Traditional Arab Islamic medicine, Palliative care, Supportive cancer care

## Abstract

This study aimed to explore integrative oncology (IO) across the Middle East, examine how international IO leaders promote IO in the Middle East, and assess how the Middle East contributes to the development of IO globally. This narrative review employed a structured literature search and descriptive analytic approach, using multiple databases, predefined keywords, varied study designs, and multilingual sources. It was conducted by a multi-disciplinary team of clinicians, researchers, and medical educators from ten Middle Eastern countries (Cyprus, Egypt, Iran, Israel, Jordan, Morocco, Palestine, Sudan, Turkey, and United Arab Emirates) and global IO/palliative leaders from South America, India, China, Europe, and the USA, providing a real-life implementation perspective. The collaborative IO model of care ensures safe and effective traditional and herbal medicine practices, with a culturally-sensitive approach respecting health beliefs. Middle Eastern and international IO participants discuss how they can benefit from each other, drawing from Middle-East Research Group in Integrative Oncology (MERGIO) activities in clinical practice, research, and medical education. Important themes derived from the review include the potential for IO to advance patient-centered care, effectively improve quality of life-related concerns, ensure a safe therapeutic environment, and overcome accessibility- and disparity-related barriers. IO treatments should be provided by trained multi-disciplinary teams of integrative physicians and practitioners, within culturally-adapted multi-modal programs and a receptive national and regional healthcare policy. Research utilizing pragmatic methodologies reflects real-world IO practice, with IO prioritized in medical education providing students and healthcare professionals with high-quality clinical and research training across the Middle East.

## Introduction

The Middle East is the home to the birth of Traditional Arab and Islamic medicine (TAIM [1]. TAIM is an important part of healthcare across the Middle East and is often used outside the conventional oncology setting, in parallel to conventional medical treatments and often without the knowledge of the patient's oncology healthcare providers (HCPs). The lack of an open dialogue between HCPs and patients can lead to significant communication barriers; mistrust of the other; non-acceptance of the recommended conventional oncology treatment regimen, with reduced adherence; and increased risk of toxicities, side-effects, and herb-drug interactions, all of which may compromise anti-cancer treatment outcomes [2].

The emerging paradigm of integrative medicine within the oncology setting, a concept termed "integrative oncology" (IO), is characterized by evidence-based complementary modalities provided within the oncology center, as an integral component of conventional supportive/palliative care [3]. For more than two decades, the field of IO has been led globally by the Society for Integrative Oncology (SIO), which has as its goal the promotion of patient-centered and evidence-informed treatments for patients with cancer [4]. The SIO has been working hand-in-hand with the American Society of Clinical Oncology (ASCO) in publishing clinical practice guidelines on the use of IO in patients with breast cancer [5]; for cancer-related pain [6]; anxiety and depression [7]; and most recently, cancer-related fatigue [8]. The SIO Global Committee has been actively promoting the implementation of these SIO-ASCO guidelines worldwide.

Despite the increasingly growing amount of state-of-the-art evidence supporting the inclusion of IO programs, in present and future directions for patient-centered cancer care, implementation goals in low- and middle-income countries (LMICs) remain unmet [9]. In 2010, a group of HCPs collaborating within the Middle East Cancer Consortium (MECC) established the Middle-East Research Group in Integrative Oncology (MERGIO). The goals of MERGIO are to explore the characteristics of IO models across the Middle East; and to facilitate the establishment of new models which have been adapted to the local health system, resources, and social-cultural-religious values. The present study explores the perceptions among leading Middle Eastern and international figures from the field of IO, creating a discussion on how each can contribute to advancing this field as it relates to clinical practice, research, and medical education. The bi-directional relationship between the MERGIO and international IO centers was studied as well.

## Methods

The present study is a narrative review, in accordance with the key steps recommended by Sukhera and with the goal of pursuing a flexible and broad perspective, using a rigorous approach in analyzing and interpreting the literature [10]. A multi-disciplinary team of twenty clinicians, researchers, and medical educators from ten Middle Eastern countries (Cyprus, Egypt, Iran, Israel, Jordan, Morocco, Palestine, Sudan, Turkey, and United Arab Emirates) and nine international centers (Brazil, China, France, Germany, India, Italy, Switzerland, UK, and U.S.) participated in the design of the study. The team of researchers included thirteen medical doctors (board-certified in family medicine, internal medicine, oncology palliative care, pediatrics, and pediatric oncology), four nurses, five PhD researchers, and nine medical educators. The primary objective of the narrative review was to map IO activity across the Middle East and to examine the participants’ response to the following two questions:

- How can the international community promote the advancement of IO in the Middle East?
- How can the Middle East contribute to the development of IO globally?

A search of the scientific literature was then conducted, using the online MEDLINE/Pubmed search engine; the Cochrane Database of Systematic Reviews; and MERGIO publications. Keywords used in the search included the following: alternative/complementary/traditional/integrative medicine; medical oncology; surgery; effectiveness, safety/risk; Traditional Arab and Islamic medicine (TAIM); and other complementary and alternative medicine (CAM) modality-specific keywords (e.g., acupuncture, touch, reflexology, mind–body, herbal medicine, nutrition, Anthroposophic medicine). Inclusion criteria comprised all of the following:

1. Explanatory studies (i.e., randomized, controlled trials; RCTs), pragmatic studies, quasi-experimental studies; meta-analyses and systematic reviews.
2. Research papers in which the first, second, or last author are with an academic affiliation in the Middle East from Morocco in the west to Iran in the east, and from Yemen and Sudan in the South to Turkey in the North.
3. Research papers on one of the following IO modalities in patients with cancer: herbal medicine, TAIM, traditional Chinese medicine (including acupuncture), and touch, movement, and mind body therapies. In cases in which it was unclear as to whether the intervention should be included or not, two independent IO researchers were consulted.

Exclusion criteria included publications reported on patients treated in regions outside the Middle East, patients without a cancer diagnosis, and/or a mixed cohort with cancer and non-cancer diagnoses (not including those patients with cancer as well as other co-morbidities).

While the search was conducted primarily on English-language publications, the researchers also reviewed papers published in Arabic, Farsi, French, Hebrew, and Turkish. Following the selection of the relevant research papers, the authors performed a descriptive analysis, assessing the relevance of the data related to the IO setting. The findings were then organized descriptively into overarching categories. Of the 174 articles identified, 157 were screened, 88 were included in the review (45 referenced in the present article), and 69 excluded due to inability to meet the study’s inclusion/exclusion criteria.

## Results

### Integrative oncology in the Middle East

Table 1 presents the scope of IO-related activities taking place across the Middle East, summarizing these activities as they relate to clinical practice, research, and medical education. TAIM use was found to be widespread among patients with cancer in Middle Eastern countries, ranging from 35% in Iran to 46% in Morocco; 51% in Israel; 57% in Turkey; 90% in Saudi Arabia; and nearly 100% in Jordan [11]. Table 1 shows a significant degree of IO clinical practice within oncology centers in Israel and Cyprus, with preliminary implementation of integrative multi-disciplinary models in Egypt, Iran, and Turkey. In Israel, ten oncology centers have an active IO service [12], with a national IO training program for physicians, nurses, and therapists currently underway, with the endorsement of the Israel Medical Association. Significant government-supported IO research and training are taking place in Iran and Turkey, with the establishment of the Office of Persian and Complementary Medicine in the Iranian Ministry of Health and Medical Education; and the Institute of Traditional and Complementary Medicine in Turkey.

**Table 1:** Integrative oncology (IO) activities in the Middle East and Northern Africa

Country (in alphabetical order)	Clinical practice	Clinical research	Medical education
Cyprus	IO department, German Oncology Centre (Limassol, est. 2020). IO services are for the most part covered by the hospital	Undergoing collaborative research projects with the SIO Global Task committee and MERGIO, exploring patients’ perspectives on developing IO models of care. Published an RCT on MBM (anxiety, pediatrics) [26]	In 2023, hosted an SIO research workshop Planned lectures to medical students (clinical years)
Egypt	TAIM practices outside oncology centers. Rates of TAIM use is 60% in pediatric oncology [27]. Unpublished MERGIO study conducted in Gharbia, Egypt, suggest that 66% of patients with cancer use TAIM/CAM. No IO model of care at present in Egypt	Most TAIM-related studies limited to herbal medicine. Published IO-related RCTs focusing on oncology treatment-related toxicities [28], including in the pediatric oncology setting [29]	No IO educational programs currently taking place
Iran	Systematic review of 604 articles found a mean of 52% for use of CAM/PTM in patients with cancer [30]. IO services provided in many of 70 TPM-oriented health centers/cancer referral hospitals, often in the form of lifestyle modification (e.g., herbal medicines, massage, and cupping)	Several CAM/TPM research centers established, including in the Department of IO and QoL at Motamed Cancer Institute (since 2014). Published QoL-focused RCTs, assessing impact on oncology treatment-related toxicities, effectiveness of herbal PTM [31], manual [32], mind–body [33], and spiritual modalities [34]	Ministry of Health and Medical Education Office Persian and Complementary Medicine Department promoting Persian medicine in faculties. Basic TPM training for HCPs is being implemented as a pilot in nine universities, with a goal of expanding to 14 universities
Israel	Rates of CAM use ranging from 40 to 60% among patients undergoing active oncology treatments, many supporting inclusion of IO services [13]. Currently ten IO programs in cancer centers nationwide [25]. Inclusion of IO in National Health Basket promoted by the Society for Complementary Medicine, Israel Medical Association	Clinical IO research including observational and RCTs, focusing on QoL-related concerns (e.g., chemotherapy-related neuropathy [35]), adherence to chemotherapy protocol, perioperative anxiety and pain [36], cross-cultural medicine, and DEI in IO care [37]	IO is included in compulsory integrative medicine curriculum in some medical faculties. Chief nurse of largest health maintenance organization acknowledged IO training syllabus developed for palliative care nurses [38]. National-scale IO training programs for physicians, nurses, and therapists underway, with support of Israel Medical Association
Jordan	Use of herbal medicine (35%) within specialized cancer center-based CAM [39]. High rates of CAM use (65%) in pediatric oncology [40]	Published studies on outlooks among physicians and patients on CAM/TAIM in cancer care [15, 41]; on integration in supportive care [22], on nurse perspectives regarding spiritual care [42], parents’ perspectives of CAM in pediatric oncology [43]	Planned inclusion of IO in training curricula of medical schools and HCP residencies
Morocco	High rates of CAM (71%) and TAIM use, primarily ISPs (60% of users) and herbal medicine [44]. IO modalities (e.g., herbal medicine, acupuncture, MM) gradually being incorporated into cancer patient care, though not yet established in specialized centers	RCTs in IO as yet unpublished	Incorporation of IO-related subjects in medical school curriculum as yet unclear
Palestine	Prevalence of CAM use (mainly herbs) is 69% among patients with cancer [45]. No IO services currently provided in oncology centers	Research about herbal use among patients with cancer and drug-herb interactions [46]	No IO educational programs currently taking place
Sudan	No IO services currently provided in oncology centers	Limited TAIM/CAM published research	No IO educational programs currently taking place
Turkey	Rates of CAM use (mainly herbs and supplements) ranging from 40 to 95% among patients with breast and gynecologic cancer. Complementary medicine practices authorized only to physicians and dentists, within clinical and academic settings. Leading academic centers and government hospitals have adopted integrative multidisciplinary models of care, including for nurses	The Institute of Traditional & Complementary Medicine of Turkey (established in 2014) focuses its clinical research on health beliefs and patient perceptions [47]; safety and efficacy of herbal medicine, touch and MBM modalities [48]	Acupuncture Treatment Regulation (1991, revised 2002) with acupuncture training curriculum. Ministry of Health-published guidelines for training and evaluation proficiencies through CTM clinical trials. IO gradually incorporated into medical and nursing school curriculum. Formal IO certification programs/training programs are limited
United Arab Emirates	CAM practices including cupping and hot sand baths. Naturopathic oncologist working with oncology team at the Burjeel Medical City (not covered by medical insurance)		

*CA* Cancer, *CAM* complementary and alternative medicine, *CTM* complementary and traditional medicine, *est.* established, *HCPs* healthcare providers, *ISPs* Islamic spiritual practices, *MBM* mind–body medicine, *Pts* patients, *QoL* quality of life, *RCT* randomized controlled trial, *SIO* Society for Integrative Oncology, *TAIM* traditional Arabic and Islamic medicine, *TPM* traditional Persian medicine

Key patterns emerging from Table 1 included the following themes:

- **Exploring patients’ expectations** regarding how to design an IO model of care, attuned to TAIM. Questionnaire-based studies have been conducted in Israel (*N* = 770) [13, 14], Palestine (*N* = 324, vs. Israeli Arabs, *n* = 324) [15], Jordan (*n* = 253) [16], Iran (*n* = 176) [17], and Turkey (*n* = 247) [18], with ongoing studies currently taking place in Cyprus, Egypt, and Palestine. These studies highlighted patients’ expectations for enhanced doctor/nurse-patient-caregiver communication, with the goal of the referral by HCPs to IO consultations and treatments within the oncology center.
- **Exploring HCP’s perceptions** regarding the implementation of the IO model within supportive cancer care [19]. This, included identifying challenges in HCP-patient communication on TAIM use [20]; and surveys of oncology HCPs in Iran (*n* = 202) [21] and Jordan (*n* = 240) [22], examining perspectives on referral to IO consultations and involvement in the design of the IO treatment program.
- **Addressing the risk/safety of IO**, particularly the potential risks associated with the use of traditional herbal medicine during conventional oncology treatments [23]
- **Medical/health education of HCPs in IO competencies**, with the goal of addressing unmet QoL-related patient concerns. HCP training was perceived in the format of hands-on clinical and research workshops, in collaboration similar to those initiated by MERGIO with Middle Eastern and European IO clinicians in Germany [24], Israel [25], and Switzerland.
- **Cross-cultural medicine**, spirituality and religion in mind–body medicine, particularly in palliative care

While Table 1 presents a discrepancy between the clinical IO-related activities between countries, where they are most prominent in Israel and Cyprus and, to a lesser degree, in Egypt, Iran, and Turkey, overarching themes were identified across countries with respect to clinical research and planned medical education programs.

### International support of IO in the Middle East

**Fig. 1:**
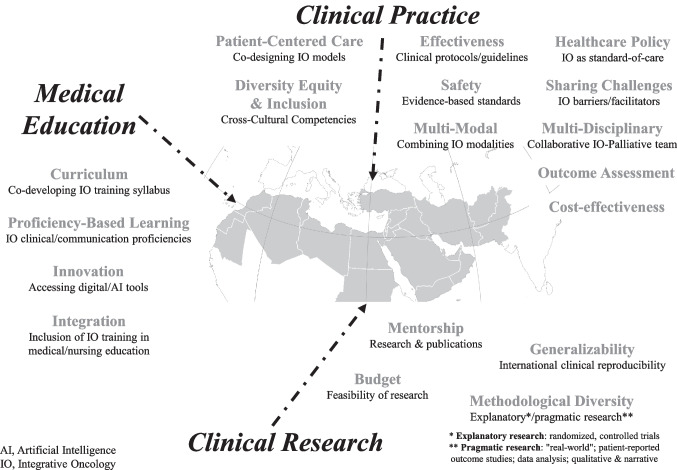
How can the international community promote the advancement of IO in the Middle-East?

Table 2 presents the overarching themes as they relate to the question: “How can the international community promote the advancement of IO in the Middle East?” The expectations among IO leaders in the Middle East and international IO centers were focused on addressing ways in which the global community could help promote the advancement of IO in the Middle East, specifically through a stronger collaboration with MERGIO. Figure 1 illustrates the main themes identified among Middle Eastern respondents, describing how they see the international IO community providing support to advance IO-related clinical practice, research, and medical education.

**Table 2:** Expectations from global mentoring and support of a collaborative Middle Eastern integrative oncology project: How can the international community promote the advancement of IO in the Middle-East?

Country (in alphabetical order)	Clinical practice	Clinical research	Medical education
**Middle-Eastern perspective** If a collaborative MERGIO project was co-designed with other global centers, in which domains would you expect to gain mentoring and support from other regions across the globe?			
Cyprus	* Adopting/implementing scientifically validated IO therapies into standard cancer care (e.g., safety-related guidelines; quality assurance) * Developing and adapting clinical guidelines tailored to the local context of care * Supporting the design of patient-centered care models; implementation of AI-driven tools to enhance patient care and personalized care	* Supporting additional research projects with mentorship and guidance to ensure the successful completion of these studies	* Assisting IO curriculum development for medical and HCP education programs (incl. cross-cultural competencies) * Organizing training workshops and seminars * Providing support in developing and accessing digital learning tools (incl. AI and data science) * Establishing Mentorship programs; facilitating mentor-trainee communication and rapport, fostering IO-related proficiencies
Egypt	* Providing IO co-training initiatives to inspire and promote the establishment of IO services in the country	* Mentoring IO research will enhance the understanding of challenges and barriers, identifying opportunities and strengthening professional acceptance of IO	* IO training for HCPs, including physicians and nurses, with the goal to establishing IO services across the country
Iran	* Developing/adopting clinical protocols/guidelines and best practices from leading IO centers focusing on effectiveness and safety * Mentoring would be invaluable in improving patient care, particularly in oncology, palliative care, and nursing * Gaining support in IO-related policy development	No perspectives provided	* Mentoring how to effectively integrate IO into medical and healthcare curricula * Learning about best practices in IO curriculum; how to implement IO programs and policy/regulatory frameworks that support the integration into mainstream medical education
Israel	* Identifying barriers and facilitators to IO clinical implementation, based on global IO models and experience * Inclusion of patient advocates' perspectives in development of IO models	* Design of collaborative international research with the goal to enhance generalizability * Budget support to facilitate research feasibility * Conducting cultural-sensitive research on patient and HCP perspectives	* Sharing global insights of IO training syllabus * Online participation of IO mentors in pre- and post-graduate IO teaching * Co-authoring medical education publications, emphasizing international perspectives
Jordan	* Sharing struggles and successes in IO field * Exposing trainees to evidence-based IO practices * Developing cultural competencies * Fostering trainee resilience and confidence	* Cooperating with international teams will facilitate researchers and HCPs to initiate IO/TAIM research; raise the standard of the quality of the research; and explore the impact of cultural beliefs on treatment preferences and outcomes	* Engaging methods of teaching, such as workshops and case discussions, as well as communication strategies can further enhance IO/TAIM training
Morocco	* Gaining expertise from advanced centers in developing IO technologies and treatment methods * Receiving guidance on developing healthcare policies tailored to local needs, while integrating global best practices	* Learning to manage large-scale projects involving multiple stakeholders, while effectively coordinating cross-border efforts * Collaborating on data analysis and the use of AI to improve diagnosis and treatment	* Accessing specialized IO training programs to enhance clinical and research skills
Palestine	Developing teamwork; sharing objectives and aims to provide high quality care	Funding opportunities are limited in Palestine, requiring international resources	Need for training for increased clinical skills, especially for non-pharmacologic therapies to address pain
Sudan	IO should be considered within palliative care	Conducting feasibility and cluster randomized studies to see effects of IO on patient outcomes	No IO educational programs currently taking place
Turkey	* Supporting IO models could foster multi-disciplinary care teams, patient-tailored treatments, and enhance safety guidance * Developing communication strategies with patients about IO safety and efficacy	* Mentoring the conduct and analysis of large-scale IO-related RCTs	* Mentors from countries with advanced IO training programs could help develop more structured and accredited training programs for HCPs (e.g., online courses and workshops to implement IO into daily practice)
**Global perspective** If a collaborative MERGIO project was co-designed with your center, in which domains would you anticipate to contribute/mentor?			
Brazil	* Promoting standardized multimodal IO treatments based on evidence-based ASCO/SIO guidelines and evidence maps * Developing a system for recording care in the centers, including assessment of cost-effectiveness benefits based on patient interviews * Implementing multimodal IO protocols in the Middle East	* Collaborating on the development and introduction of a system for evaluating care models using Practice Based Research Network (PBRN), centered on the patient perspective in partnership with MERGIO * Co-designing multicenter clinical trials of innovative treatments; conducting systematic reviews and publications	No perspectives provided
China	* Sharing clinical experience and research of the IO models developed in China	* Mentoring IO research focused on traditional medicine, effectiveness, and safety	No perspectives provided
Germany	* Developing day clinic programs; pre- and prehabilitation concepts; implementation of external applications; integration of IO in pediatric oncology	* Collaboration between oncology centers; contributing to studies focusing on mind–body medicine, integrative oncology, and external applications	* Providing training programs in mind–body medicine and IO; sharing clinical experiences along with various methodological approaches derived from different whole medical systems; creating opportunities to develop courses in nutritional medicine and integrative medicine as part of the clinical curriculum
India	No perspective provided	* Considering shared challenges of integrating traditional medicine within cancer care in India and the Middle East * Collaborating in order to enhance the development of clinical practice guidelines in cancer care [49]	No perspectives provided
Italy	* Collaborating with ARTOI, introducing specific IO modalities such as acupuncture, homeopathy and European herbal medicine	No perspectives provided	No perspectives provided
Switzerland	* Promoting the work of MERGIO, increasing its international visibility and recognition	No perspectives provided	* Training and mentoring on how to advocate and promote IO at the health policy level
United Kingdom	* IO mentoring in a multimodal and multidisciplinary team, with an intersection between IO and precision oncology and immuno-oncology	* Sharing experience with real-world data and patient-reported outcome measurements collection, as well as literature reviews and guideline development	* Offering training programs for nutrition and medical professionals in IO

AI, artificial intelligence; ARTOI (The Italian Association in Integrative Oncology); HCP, healthcare providers; IO, integrative oncology; MERGIO, Middle East research group in integrative oncology; RCT, randomized controlled trials; SIO, Society for integrative oncology; TAIM, traditional Arab Islamic medicine

Key findings emerging from Table 2 included the following themes:

- **Clinical practice**: Enhancing patient-centered care, multi-disciplinary teamwork, and providing effective and safe evidence-based therapies, which resonate with patients’ expectations and health beliefs.
- **Clinical research**: Employing diverse research methodologies (i.e., pragmatic studies) that enable researchers to explore the effects of “real-world” daily IO practice in the Middle East. Research focused on healthcare policy, budget, and cost-effectiveness.
- **Medical education:** Mentoring Middle Eastern researchers and medical educators within nationwide training programs.

### Bidirectional relationship: collaboration between Middle Eastern and international IO centers

**Fig. 2:**
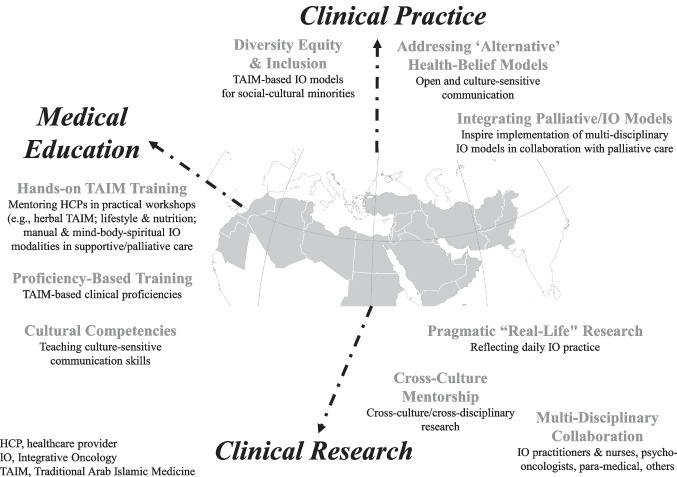
How can the Middle East contribute to the development of IO globally?

Table 3 presents the overarching themes as they relate to the question: “How can the Middle East contribute to development of IO globally?” It summarizes expectations among participants from the Middle East and leading international IO figures regarding how the global community can benefit from the activities of Middle Eastern centers and the activities of MERGIO. Figure 2 illustrates the main themes identified from the narratives of respondents from both groups (Middle Eastern and international), reflecting the “strengths” of IO in the Middle East from which the others can benefit. The key themes identified in Table 3 included the following:

- **Clinical practice:** Focus on TAIM-based IO models, which are important outside the Middle East in countries with large immigrant populations from these countries, communication skills which are important when treating patients with alternative or non-conventional health beliefs, and multi-disciplinary models of combined IO and palliative care, as practiced in many cancer centers across the region.
- **Clinical research:** Potential for mentorship in cross-cultural and multi-disciplinary aspects of IO programs, as well as experience in the design and implementation of pragmatic “real-life” research, reflecting daily IO practice.
- **Medical education:** Mentoring skills of IO teachers in the Middle East, most notably on the use of TAIM in palliative care and IO nursing, and cultural competencies within the broader context of traditional systems of medicine, as practiced within the modern hi-tech setting of contemporary oncology.

**Table 3:** Expectations of potential impact of Middle Eastern IO projects on professional growth of other global IO programs: How can the Middle East contribute to development of IO globally?

Country (In alphabetical order)	Clinical practice	Clinical research	Medical education
**Middle-Eastern perspective** Considering that collaboration between MERGIO and IO global centers is bi-directional, in which domains would you expect to contribute knowledge, attitudes, and/or skills that may facilitate professional growth in these centers?			
Cyprus	MERGIO’s experience in implementing cultural-sensitive IO practices (e.g., cultural values, religious beliefs, healthcare policies, etc.) may be valuable to global centers seeking to expand IO in multicultural settings	MERGIO could contribute research expertise in the safe and evidence-based integration of regional herbal therapies into cancer care	Organizing hybrid workshops and training to help HCPs expand their knowledge
Egypt	No perspectives provided	MERGIO can use its research experience to advance international IO research	No perspectives provided
Iran	Validating and adopting clinical protocols and guidelines to provide specific IO therapies within palliative care services	* Using qualitative, quantitative, and mixed-methods methodologies to address critical questions in IO research * Writing research papers and reports, disseminating findings through peer-reviewed journals * Fostering cross-disciplinary and cross-cultural research initiatives, including research specific to Iranian patients, comparing findings to those of global data * Establishing collaborative IO research initiatives	No perspectives provided
Israel	* Mentoring the design of IO models tailored to the characteristics, needs, expectations, and resources of the cancer care center * Mentoring a patient-centered & tailored IO program to patients with affinity to traditional health systems, including medicinal herbs	* Launching IO registry protocols as a basis for pragmatic research reflecting daily real-practice IO service *Mentoring RCTs with a patient-preference component, respecting the patient’s choices and autonomy * Guidance on IO qualitative research and narrative-based publications	* Offering multi-disciplinary IO training focused on evidence-based herbal, touch, and mind–body modalities focused on QoL-related concerns * Co-designing IO training syllabus with global academic centers for medical students and nurses *Co-designing IO national syllabus and training for HCPs from multi-disciplinary background * Mentoring HCPs with open and non-judgmental communication skills, including with patients using traditional/herbal medicine
Jordan	No perspectives provided	No perspectives provided	Sharing experiences in integrating traditional practices with modern oncology will facilitate the education of HCPs globally, while addressing cultural competence and patient-centered care
Morocco	* Sharing insights on how traditional (e.g., herbal) and modern IO (e.g., mind–body) approaches are applied in clinical oncology settings in Morocco * Offering expertise on how to navigate cultural and religious considerations in patient-centered care	* Engaging in joint research initiatives that explore the effectiveness and safety of IO treatments * Helping build a robust evidence base for these practices globally	* Providing perspectives on educational strategies and training programs that integrate CAM into medical curricula, aimed at fostering a comprehensive understanding of IO among HCPs
Palestine	No perspectives provided	No perspectives provided	Adding a nursing perspective
Sudan	Contribution in clinical practice of holistic patient assessment and management	No perspectives provided	Contributing to training of multi-disciplinary healthcare professionals
Turkey	Turkey’s unique location at the crossroads of East and West allows for a wealth of experience in balancing traditional and modern treatments. Sharing this knowledge could improve global understanding of culturally sensitive IO treatments, especially in countries with a distinct spiritual/religious culture	* Supporting the design of cross-cultural research * Turkey’s research expertise could enhance global research, addressing how cultural contexts influence the adoption and effectiveness of IO treatments * Turkey’s experience with the use of herbal medicine in oncology could be valuable in collaborative research on herb-drug interactions and safety guidelines	Turkey’s growing multidisciplinary approach to oncology care, with a tradition of integrating holistic and spiritual practices, could provide valuable insight for other countries
**Global perspective** Would you expect any professional growth in your own practice resulting from a collaboration with MERGIO?			
Brazil	MERGIO would be able to support and inspire the Latin America and Brazilian Strategic Plan in partnership with CABSIN, PAHO/WHO, SIO, TCIM America Network, and MERGIO. This may facilitate high-quality scientific publications of IO, creating clinical guidelines and promoting equitable knowledge translation actions	Collaboration with MERGIO may inspire surveys of IO models of care in Latin American cancer centers exploring patients’ perceived care in partnership with SIO, LARGIO, MERGIO, CABSIN and BraveNet	A strategic plan could be created to promote an extensive, evidence-based, standardized, interdisciplinary integrative oncology postgraduate program in collaboration with the SIO, MERGIO, and academic oncology centers
China	Exploring the IO models in Beijing based on the insights and experience of the MERGIO IO models	Potential collaboration in research synthesis for creating clinical guidelines and clinical reference	Potential for medical education collaborations
Germany	Joint development of care models and interventions for different oncology subdisciplines	Conducting clinical trials within international research networks; including surveys and multicenter studies, as well as meta-analyses and reviews	Collaborative development of training programs, guidelines and quality assurance in IO
India	MERGIO’s experience may provide valuable insights on how Ayurveda can be incorporated into IO practice in India, identifying important aspects for integration of Ayurveda in oncology care MERGIO's input may enhance the development of evidence-based guidelines for the clinical practice of oncology in India	A collaboration with MERGIO would help in designing proper studies to evaluate the efficacy of Ayurveda in cancer care	Collaboration in medical education may enhance the design of IO training programs for HCPs
Italy	No perspectives provided	Professional dialogue between MERGIO and European IO associations in could enhance clinical research whose goal is to improve quality of life, and enhance international growth of integrative medicine	Organizing hybrid workshops and training to help HCPs expand their knowledge
United Kingdom	* Exposing others to Middle Eastern IO practices, many of which may be unfamiliar * Learning more about cross-cultural practices and IO implementation models on the ground, and how these are tailored to the local environment and needs	Research collaborations with MERGIO	Exposure to different training models and teaching approaches would prove invaluable in developing and refining teaching styles, expanding the educational tool kit

*CAM* complementary and alternative medicine, *HCP* healthcare providers, *IO* integrative oncology, *MERGIO* Middle East research group in integrative oncology, *QoL* quality of life, *RCT* randomized controlled trials, *SIO* Society for integrative oncology

## Discussion

The present narrative review suggests that IO in the Middle East can play an important role in advancing clinical practice worldwide, including in research and medical education, most notably for the treatment of oncology patients who have an affinity for traditional medicine practices, TAIM in particular. This is important in light of the fact that the overwhelming majority of established IO programs are in Western countries. The exception to this rule is countries such as China, Japan, and South Korea, where traditional Chinese and Oriental medicines are part of conventional medical systems, and aligned with patients’ preferences and beliefs. The rapid increase in the establishment of IO services in the West reflects the recognition of evidence-based research by the medical community, as cited by the collaborative SIO-ASCO clinical practice guidelines. In contrast, the core values of traditional medicine in other regions of the globe, including the Middle East, continue to perceive modalities such as herbal and dietary supplement use as “crude herbs,”herbs’, with “mind-body medicine” practiced within a spiritual-religious context.

It is within this context that IO settings in the Middle East represent an intermediary “go-between” role on the continuum between West and East, North and South. This is in keeping with the historic role of the Middle East as a birthplace for Western medicine, originating as a center for the confluence of Ayurveda and traditional Chinese medicine with Ancient Greek medicine. The Middle East can thus serve as a bridge, connecting the highly evidence-based IO practices of the West with the more experience-based traditional medicine of the rest of the world, whether in Africa, South America, Asia, or even minority immigrant and refugee groups, from Oslo to New York.

The present narrative review highlights the importance of the contribution of the MERGIO collaboration in clinical practice, research, and medical education. This is even more important in light of this uniquely diverse region, which is saturated with armed conflicts and in which scientific collaboration is often not possible between Middle Eastern countries due to real and often existential safety and political risks to clinicians, researchers, and medical educators who wish to collaborate with each other. These challenges are largely being overcome through the shared commitment to the welfare of patients with cancer, whether Turkish, Egyptian, Palestinian, Israeli, Lebanese, Iranian, or citizens of the other Middle Eastern countries. When collaboration between countries with geopolitical divisions occurs, the support and non-partisan platform provided by colleagues outside the Middle East is needed to enable this fragile miracle to continue and prosper. The aspiring gesture to facilitate IO in the Middle East is reflected by the diversity of the co-authors of the present narrative review, who together with those from outside the Middle East embrace this *alchemical flame*, encouraging it to continue and spread.

In order for the ongoing collaboration between MERGIO and others in the Middle East with international IO organizations to expand and prosper, the World Health Organization should be encouraged to support the establishment of a Center of TAIM-based IO Excellence in the Middle East. Such an endeavor would significantly help promote the goal of advancing IO in the region, through hands-on training workshops which would take place within and outside the Middle East. Workshops such as these would provide multi-disciplinary training to teams of HCPs, implementing IO modalities and techniques (e.g., touch-movement, mind–body, herbal-nutritional based on the Middle Eastern cuisine) which can help alleviate specific QoL-related concerns among patients throughout their cancer journey. This medical education initiative would be led by MERGIO, and would be followed by the establishment of IO Centers of Excellence in at least one oncology center in each of the participating MERGIO countries. The practical design and establishment of innovative IO programs will hopefully be supported by mentorship from other international IO centers, as suggested in Fig. 1. This may be followed by a bi-directional international collaboration, as suggested in Fig. 2, which would include IO educators from the Middle East providing mentorship to other IO centers worldwide.

The present narrative review has many methodological limitations, and reflects only a portion of the extensive published IO literature, with subjective interpretation by the authors. While acknowledging these limitations, we do believe that the strength of this review is in its courage and commitment to advancing patient care by IO clinicians, researchers, and educators who prioritize collaboration over isolation, with a profound commitment to the spirit of healing.

## Data Availability

Data transparency is available pending to request from the submitting author.
